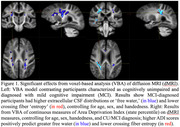# MCI Diagnosis and Neighborhood Disadvantage Predict Differences in White Matter Microstructure in a Rural Aging Cohort

**DOI:** 10.1002/alz.094061

**Published:** 2025-01-09

**Authors:** Andrew R. Bender, Pavithran Pattiam Giriprakash, Dietmar Cordes, Xiaowei Zhuang, Jessica ZK Caldwell, Justin B Miller

**Affiliations:** ^1^ Cleveland Clinic Lou Ruvo Center for Brain Health, Las Vegas, NV USA; ^2^ Nevada Exploratory Alzheimer’s Disease Research Center, Las Vegas, NV USA

## Abstract

**Background:**

Diffusion magnetic resonance imaging (dMRI) studies show cerebral white matter alterations are associated with both mild cognitive impairment (MCI) and contextual psychosocial factors. However, these reports are almost exclusively based on older adults residing in metropolitan areas. Individuals living in rural communities face increased risk for Alzheimer’s disease and related dementias, but little is known regarding the sensitivity of dMRI markers to incident MCI or psychosocial stressors in this population. In a rural‐dwelling community cohort of older adults, this study evaluated associations between dMRI measures of white matter microstructure and both clinical MCI diagnosis and neighborhood disadvantage.

**Method:**

The study sample included rural‐dwelling older adults (n = 69) from the Nevada Exploratory Alzheimer’s Disease Research Center. Participants were characterized as cognitively unimpaired (CU; n = 42) or diagnosed with MCI (n = 27) based on clinical expert consensus, and all had dMRI data and Area Deprivation Index (ADI) measures of neighborhood disadvantage. dMRI data processing leveraged the MRtrix3 fixel‐based analysis framework and free water mapping to estimate voxel‐based measures of white matter organization, transformed to a sample template. Data analysis utilized voxel‐based analysis (VBA) to model differences in clinical diagnosis and ADI scores as predictors of fiber density, crossing fiber entropy, and extracellular free water, and free water‐corrected diffusion tensor parameters, accounting for differences in age, sex, and handedness.

**Results:**

The VBA models showed both MCI diagnosis and elevated neighborhood disadvantage predicted significantly greater (familywise error corrected p<.05) free water and reduced crossing fiber entropy in centrum semiovale and internal capsule (Figure 1). Uncorrected models for both diagnosis and higher ADI level revealed widespread associations with dMRI measures of free water, tensor parameters, and fiber density that were nonsignificant following permutation‐based corrections.

**Conclusion:**

These preliminary findings reveal white matter alterations associated with consensus MCI diagnosis and to socioeconomic differences in a clinical cohort of rural‐dwelling older adults. The present results highlight the value of modeling extracellular cerebral spinal fluid or ‘free water’ as a marker of microstructural changes associated with both cognitive impairment and contextual psychosocial factors. Future efforts are needed to compare these effects between rural‐ and metropolitan‐dwelling older adults at risk for dementia.